# Retrospektive Mortalitätsstudie natürlicher Todesfälle der Generation 65+ im Obduktionsgut der Rechtsmedizin Frankfurt am Main anhand zweier Zeitintervalle

**DOI:** 10.1007/s00194-021-00469-6

**Published:** 2021-03-08

**Authors:** A. Wach, C. Faßbender, H. Ackermann, M. Parzeller

**Affiliations:** 1 Institut für Rechtsmedizin, Universitätsklinikum, Goethe-Universität Frankfurt a.M., Kennedyallee 104, 60596 Frankfurt am Main, Deutschland; 2 Institut für Biostatistik und Mathematische Modellierung, Zentrum der Gesundheitswissenschaften, Klinikum und Fachbereich Medizin, Goethe-Universität Frankfurt a.M., Frankfurt am Main, Deutschland

**Keywords:** Todesart, Kardiale Todesursachen, Sektion, Geriatrie, Gerontologie, Manner of death, Cardiac causes of death, Autopsy, Geriatrics, Gerontology

## Abstract

**Hintergrund und Ziel der Arbeit:**

In Deutschland vollzieht sich ein stetiger demografischer Wandel, welcher zu einer zunehmenden Alterung der Gesellschaft führt. Ziel der Arbeit war die Analyse der natürlichen Todesfälle mit einem Sterbealter ≥ 65 Jahre, da die gesundheitliche Vulnerabilität dieser Altersgruppe an Bedeutung gewinnt.

**Material und Methoden:**

Retrospektiv wurden die Obduktionsgutachten aller natürlichen Todesfälle der ≥ 65-Jährigen im Institut der Rechtsmedizin des Universitätsklinikums der Goethe-Universität Frankfurt am Main in einem Zeitvergleich (Zeitraum I: 2000–2002; Zeitraum II: 2013–2015) ausgewertet.

**Ergebnisse:**

In den Zeiträumen I und II wurden insgesamt 1206 Obduktionen in dieser Altersgruppe ermittelt. Davon wiesen 404 (33,5 %) eine nichtnatürliche Todesart auf, in 39 Fällen (3,2 %) lag eine Kombination aus natürlichem und nichtnatürlichem Tod vor, und in 94 Fällen (7,8 %) war die Todesart unklar. Die Mehrheit (*n* = 669; 55,5 %) verstarb an einer natürlichen Todesart. Die größte Gruppe davon (*n* = 350; 52,3 %) betraf kardiale Todesursachen, gefolgt von 132 (19,7 %) respiratorischen und 47 (7,0 %) abdominellen Todesursachen. Zudem lagen 37 (5,5 %) maligne Neoplasien, 37 (5,5 %) sonstige natürliche Todesursachen, 33 (4,9 %) Rupturen großer Gefäße und 33 (4,9 %) zerebrale Todesursachen vor. Im Vergleich der Zeiträume I und II fiel eine signifikante Abnahme der kardialen Todesursachen auf. Es kam insbesondere zu einer signifikanten Abnahme der hochgradigen bis verschließenden Koronarsklerosen. Zwischen beiden Geschlechtern zeigten sich signifikante Unterschiede. So wiesen Männer signifikant mehr Bypässe, Stents und Herznarben auf und erlitten ca. 10 Jahre vor den Frauen einen Myokardinfarkt.

**Diskussion/Schlussfolgerung:**

Die Ergebnisse decken sich größtenteils mit der Literatur. Die Abnahme kardialer Todesursachen könnte auf eine zunehmend bessere medizinische Versorgung und eine signifikant zunehmende Implantationsrate von Stents zurückzuführen sein. Die Rolle der forensischen Gerontologie wird –gerade in Pandemiezeiten– zunehmend an Bedeutung gewinnen.

## Abstract (en)

**Background and aim of the study:**

In Germany, a constant demographic change is taking place, which leads to an increasing aging of the society. The present study aimed to analyze natural deaths occurring at an age of ≥ 65 years, since health vulnerability in this age group is gaining importance.

**Material and methods:**

Autopsy reports of the Institute of Forensic Medicine, University Hospital of the Goethe University Frankfurt/Main, Germany, were retrospectively evaluated regarding natural death cases of ≥ 65-year-olds in a time comparison (period I: 2000–2002; period II: 2013–2015).

**Results:**

During both periods, a total of 1206 autopsies concerning this age group were performed. Among these, 404 cases (33.5%) of unnatural death and 39 cases (3.2%) of a combination of natural and unnatural death were recorded; in 94 cases (7.8%), the manner of death could not be elucidated. The majority (*n* = 669; 55.5%) included cases of natural death. In the largest group of these (*n* = 350; 52.3%), cardiac causes of death were predominant, followed by 132 (19.7%) respiratory and 47 (7.0%) abdominal causes of death. In addition, 37 (5.5%) cases of malignant neoplasms, 33 (4.9%) of ruptures of large vessels, 33 (4.9%) of cerebral, and 37 (5.5%) other cases of natural death were noted. A significant decrease of cardiac causes of death was observed in the comparison of periods I and II. In particular, there was a significant decrease in high-grade occlusive coronary sclerosis. Moreover, there were significant differences between both sexes. Men had significantly more bypasses, stents and heart scars and suffered a myocardial infarction about 10 years earlier than women.

**Conclusion:**

The results of the present study are largely consistent with the literature. The decrease in numbers of cardiac deaths may be attributed to increasingly better medical care and to a significantly higher rate of stent implantation. Especially in times of pandemics, the role of forensic gerontology will become more important.

## Einleitung

Der demografische Wandel in Deutschland steht zunehmend im Fokus der öffentlichen Diskussion. Der Anteil der jüngeren Menschen sinkt, wohingegen der Anteil älterer Menschen stetig zunimmt. Die drastische Verschiebung der demografischen Kurve verdeutlicht eine Alterung der Gesellschaft mit ihren Auswirkungen u. a. auf das Gesundheits- und Rentensystem. In der aktuellen SARS-CoV-2-Pandemie zeigt sich, dass eine höhere Lebenserwartung mitunter mit einer hohen Letalität der älteren Menschen bei dieser Pandemie assoziiert sein kann [7, 10, 15, 18, 45, 65, 66, 69]. Inwieweit die aktuelle SARS-CoV-2-Pandemie die bisher steigende Lebenserwartung negativ beeinflusst, bleibt abzuwarten.

Nach Angabe des Statistischen Bundesamtes ist heute jede fünfte Person in Deutschland älter als 66 Jahre [83]. Die Altersklasse der Personen ab 60 Jahren hat sich zwischen 1990 und 2019 von 20,4 auf 28,5 % erhöht. Es ist davon auszugehen, dass die Anzahl der Menschen in hohem Lebensalter weitersteigen wird [83]. Dabei erreichen neben dem weiblichen Geschlecht auch zunehmend mehr Männer ein höheres Lebensalter.

Aufgrund der dramatischen Rückgänge bei den klinisch-pathologischen Obduktionen etablieren sich rechtsmedizinische Obduktionsstudien zu natürlichen Todesfällen u. a. auch im Hinblick auf Ursachen bei Erkrankungen und klinischen Fragestellungen als Goldstandard. Die aktuelle SARS-CoV-2-Pandemie verdeutlicht die Relevanz rechtsmedizinischer Obduktionen zu Krankheitsverläufen etc., weil durch die organpathologische Beurteilung der Todesursachen die Pathogenese von Erkrankungen besser verstanden werden kann.

Mit zunehmendem Alter steigt der Grad an Gebrechlichkeit, Multimorbidität und Schutzbedürftigkeit. Erkrankungen können sich mit zunehmendem Alter atypisch präsentieren und sind daher häufig schwer zu diagnostizieren.

Der Tod einer Person in hohem Lebensalter ist wahrscheinlicher, Todesart und -ursachen dürfen jedoch nicht ungeklärt bleiben. Dafür werden Obduktionen benötigt, die jedoch mit zunehmendem Sterbealter seltener durchgeführt werden [25, 26, 34, 73]. Zwischen Todesbescheinigungen und per Obduktion festgestellter Todesart und -ursache bestehen nur geringe Übereinstimmungsraten [22, 26, 29, 30, 52, 53, 76, 79, 99].

Diese Problematik unterstreicht die Notwendigkeit von Obduktionen gerade im Bereich der forensischen Gerontologie, insbesondere vor dem drastischen Rückgang der Obduktionsraten in der Pathologie [82], aber auch in der Rechtsmedizin [49, 50]. Obduktionen dienen mittels pathomorphologischer Befunde dem Verständnis von nicht zuletzt noch unbekannten Krankheitsbildern.

Einschränkend ist jedoch zu berücksichtigen, dass rechtsmedizinische Obduktionen primär dem Erkenntnisgewinn und zur Klärung rechtlicher Fragestellung im Rahmen von Ermittlungsverfahren dienen, die durch die Ermittlungsbehörden/Gerichte veranlasst wurden, sodass eine gewisse Vorselektion entsteht. Gleichwohl lassen sich aus rechtsmedizinischen Obduktionsstudien insbesondere bei höheren Fallzahlen wertvolle Rückschlüsse gerade für natürliche Todesursachen ziehen.

Während sich die Studie Faßbender et al. [21] mit den nichtnatürlichen Todesfällen befasst, liegt der Fokus dieser Studie auf den natürlichen Todesarten. In diesem Obduktionsgut machten natürliche Todesarten den weitaus größten Anteil aus. In dieser Studie sollte analysiert werden, wie sich die Verteilung der natürlichen Todesursachen der Generation 65+ darstellt.

Für ein größeres Fallkollektiv und um mögliche Veränderungen über die Zeit zu detektieren, erfolgte eine Bestandsaufnahme über 2 Zeiträume.

## Material und Methoden

Die retrospektive Auswertung basiert auf den Daten der Obduktionsprotokolle und -gutachten des Institutes für Rechtsmedizin des Universitätsklinikums der Goethe-Universität Frankfurt am Main. In die Datenerhebung wurden zudem Informationen aus Todesbescheinigungen, polizeilichen und staatsanwaltschaftlichen Ermittlungsakten, Krankenhausunterlagen und histologischen Zusatzuntersuchungen einbezogen, soweit diese vorlagen.

Als Einschlusskriterien für diese Studie wurden sowohl ein Lebensalter von mindestens 65 Jahren, eine natürliche Todesart als auch das Vorliegen eines Obduktionsprotokolls festgelegt. Es wurde ein insgesamt 6‑jähriger Analysezeitraum für die Jahre 2000–2002 (Zeitraum I) und 2013–2015 (Zeitraum II) gewählt. Die erhobenen Daten wurden mit Microsoft Excel in tabellarischer Form zusammengefasst. Die statistische Signifikanz wurde mittels Chi-Quadrat-Test unter Zuhilfenahme des Programmes BiAS (Biometrische Analyse von Stichproben) berechnet [1]. Dabei galten *p*-Werte < 0,05 als signifikant und wurden mit * gekennzeichnet. Das Studienkonzept bestand in einer explorativen-deskriptiven Analyse, sodass bei der Auswertung keine multiplen Tests (Bonferroni, Bonferroni-Holm) berücksichtigt wurden. Dies erfolgte in der Absicht, keine evtl. vorhandenen Unterschiede zu übersehen. Aufgrund des explorativen Designs konnte keine Fallzahlberechnung erfolgen. In diesem Sinn werden die errechneten *p*-Werte im deskriptiven Sinn verwendet.

Für eine Vergleichbarkeit zwischen den Zeiträumen I und II wurde Pearsons Assoziationskoeffizient phi und zur Interpretation die Bewertung nach Cohen [1, 38] verwendet. Hierbei galt phi ≈0,1 als „small“, phi ≈0,3 als „medium“ und phi ≈0,5 als „large“. Somit konnten Unterschiede zwischen beiden Zeiträumen als statistisch „schwach relevant“, „mäßig relevant“ oder „stark relevant“ gewertet werden.

Der Fokus wurde auf die Subgruppenanalyse der kardialen Todesfälle (Subgruppe (*n* = 350)) innerhalb der Gruppe der natürlich Verstorbenen (*n* = 669) gelegt. Dabei wurde der Begriff der kardialen Todesfälle weit gefasst, worunter etwa (akute) Myokardinfarkte, Reinfarkte, Herzbeuteltamponaden und -insuffizienzen fielen. Diese wurde der Vergleichsgruppe aller natürlich Verstorbenen, welche keine rein kardiale Todesursache aufwiesen (Vergleichsgruppe (*n* = 319)) gegenübergestellt. Ausgewertet wurden folgende Parameter: Todesart, Todesursache nach Organsystem, Alter und Geschlecht, Todeszeitpunkt, Body-Mass-Index (BMI), Gefäßstatus, Diabetes mellitus und Alkoholabusus sowie herzspezifische Befunde. Besonderes Augenmerk wurde auf den Vergleich beider Geschlechter und der erhobenen Zeiträume gelegt. Bei den Begleiterkrankungen Diabetes mellitus und Alkoholabusus muss einschränkend erwähnt werden, dass keine einheitliche Datenlage vorlag, da Vorbefunde nur teilweise vorhanden waren. Folglich war eine Bewertung nur eingeschränkt möglich.

Das Studienkollektiv wurde in 4 Altersklassen von je 10 Jahren eingeteilt: Klasse eins 65 bis 74 Jahre, Klasse zwei 75 bis 84 Jahre, Klasse drei 85 bis 94 Jahre und Klasse vier ≥ 95 Jahre. Für Parameter, welche spezifische Lokalisationen beinhalteten (wie z. B. die betroffene Myokardwand bei Myokardinfarkt) wurde ein Punktesystem angelegt, um bei Doppelnennung eine einheitliche Erfassung zu ermöglichen. Eine Mehrfachnennung pro Personenfall war damit möglich.

Ein Votum der zuständigen Ethikkommission lag vor (Ethikkommission des Fachbereichs Medizin der Goethe-Universität Frankfurt am Main, Nr. 116/14).

## Ergebnisse

### Todesart

**Figure 1:**
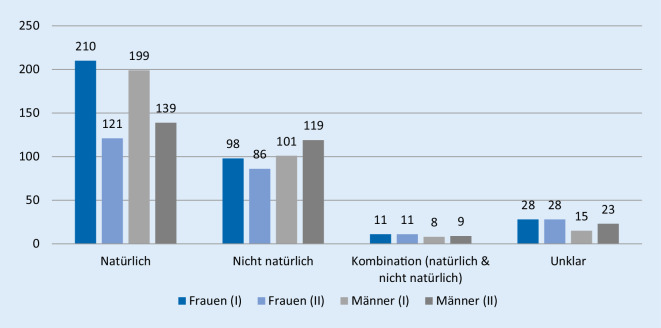

Im untersuchten Zeitraum wurden 1206 Obduktionen ermittelt. In Abb. 1 sind die Todesarten nach Geschlechtern und Zeiträumen (I, II) aufgeteilt.

Trotz der sinkenden Obduktionsraten (Zeitraum I = 670; Zeitraum II = 536) zeichnete sich im Vergleich beider Zeiträume eine deutliche Abnahme der natürlichen Todesfälle um 12,5 % ab (*p* = 0,000; phi = 0,125). Von den 669 natürlichen Todesfällen wurden 409 Fälle (61,0 %) im Zeitraum I, jedoch lediglich 260 Fälle (48,5 %) im Zeitraum II ermittelt.

Unter allen per Obduktion festgestellten natürlichen Todesarten wurde im Leichenschauschein am häufigsten die Todesart „ungeklärt“ angekreuzt (*n* = 430; 64,3 %; Zeitraum I: *n* = 284; 69,4 %, Zeitraum II: *n* = 146; 56,2 %), gefolgt von fehlenden Angaben zur Todesart (*n* = 126; 18,8 %; Zeitraum I: *n* = 93; 22,7 %, Zeitraum II: *n* = 33; 12,7 %). In 63 Fällen (9,4 %; Zeitraum I: *n* = 2; 0,5 %, Zeitraum II: *n* = 61; 23,5 %) wurde die Todesart „natürlich“ angekreuzt, hier lagen jedoch Besonderheiten wie z. B. Fäulnis oder ein vorangegangener Sturz vor. Der Verdacht auf eine „nichtnatürliche Todesart“ wurde in 50 Fällen (7,5 %; Zeitraum I: *n* = 30; 7,3 %, Zeitraum II: *n* = 20; 7,7 %) dokumentiert.

### Todesursachen

**Figure 2:**
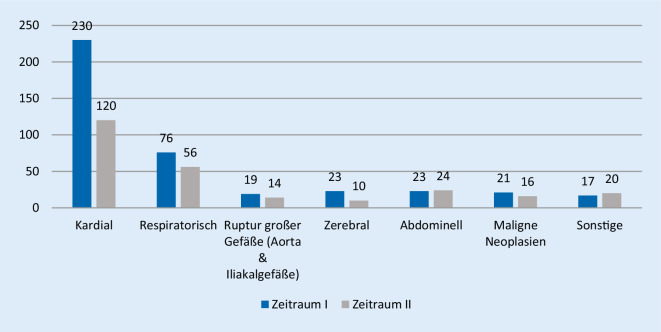

Die Todesursachenverteilung nach Organsystem innerhalb der Untersuchungsgruppe der natürlich Verstorbenen ist in Abb. 2 dargelegt. Hierbei war bei 350 Verstorbenen (52,3 %) der Tod primär kardial bedingt. Im Zeitvergleich ließ sich eine signifikante Abnahme (*p* = 0,014; phi = 0,098) der rein kardialen Todesursachen verzeichnen.

### Alter und Geschlecht

Innerhalb der Subgruppe wiesen signifikant mehr Männer (*n* = 111; 57,2 %; *p* = 0,000) in der ersten Altersklasse eine primär kardiale Todesursache auf. Signifikant mehr Frauen (*n* = 75; 48,1 %; *p* = 0,001) verstarben erst in der zweiten Altersklasse.

In der Vergleichsgruppe starben signifikant mehr Männer (*n* = 74; 51,4 %; *p* = 0,000) in der ersten Altersklasse, wohingegen signifikant mehr Frauen (*n* = 63; 36 %; *p* = 0,001) in der dritten Altersklasse verstarben.

Das Durchschnittsalter aller natürlich Verstorbenen (Subgruppe + Vergleichsgruppe) lag im Zeitraum I bei 77,6 Jahren, im Zeitraum II mit 77,7 Jahren etwas höher. Weitere Ergebnisse sind in Tab. 1 zusammengefasst.

**Table 1:** 

	Subgruppe	Vergleichsgruppe	*p*-Werte
Durchschnittsalter (Jahre)	76,9	78,4	–
Alter: Median (Jahre)	76	76	–
Alter: Minimum (Jahre)	65	65	–
Alter: Maximum (Jahre)	97	100	–
Altersklasse 1, *n* (%)	149 (42,6)	124 (38,9)	0,371
Altersklasse 2, *n* (%)	134 (38,3)	103 (32,3)	0,124
Altersklasse 3, *n* (%)	65 (18,6)	89 (27,9)	0,006*
Altersklasse 4, *n* (%)	2 (0,6)	3 (0,9)	0,673
Zeitraum I: Altersklasse 1, *n* (%)	99 (43,0)	69 (38,5)	0,415
Zeitraum I: Altersklasse 2, *n* (%)	84 (36,5)	66 (36,9)	1,000
Zeitraum I: Altersklasse 3, *n* (%)	46 (20,0)	41 (22,9)	0,555
Zeitraum I: Altersklasse 4, *n* (%)	1 (0,4)	3 (1,7)	0,323
Zeitraum II: Altersklasse 1, *n* (%)	50 (41,7)	55 (39,3)	0,792
Zeitraum II: Altersklasse 2, *n* (%)	50 (41,7)	37 (26,4)	0,014*
Zeitraum II: Altersklasse 3, *n* (%)	19 (15,8)	48 (34,3)	0,001*
Zeitraum II: Altersklasse 4, *n* (%)	1 (0,8)	0 (0,0)	0,462
Männer, *n* (%)	194 (55,4)	144 (45,1)	0,001*
Durchschnittsalter (Jahre)	74,6	75,9	–
Alter: Median (Jahre)	76	76	–
Alter: Minimum (Jahre)	65	65	–
Alter: Maximum (Jahre)	97	93	–
Altersklasse 1, *n* (%)	111 (57,2)	74 (51,4)	0,340
Altersklasse 2, *n* (%)	59 (30,4)	44 (30,6)	1,000
Altersklasse 3, *n* (%)	23 (11,9)	26 (18,1)	0,149
Altersklasse 4, *n* (%)	1 (0,5)	0 (0,0)	1,000
Zeitraum I, *n* (%)	117 (50,9)	82 (45,8)	0,360
Zeitraum II, *n* (%)	77 (64,2)	62 (44,3)	0,002*
Frauen, *n* (%)	156 (44,6)	175 (54,9)	0,001*
Durchschnittsalter (Jahre)	79,7	80,4	–
Alter: Median (Jahre)	76	76	–
Alter: Minimum (Jahre)	65	65	–
Alter: Maximum (Jahre)	95	100	–
Altersklasse 1, *n* (%)	38 (24,4)	50 (28,6)	0,459
Altersklasse 2, *n* (%)	75 (48,1)	59 (33,7)	0,011*
Altersklasse 3, *n* (%)	42 (26,9)	63 (36,0)	0,098
Altersklasse 4, *n* (%)	1 (0,6)	3 (1,7)	0,626
Zeitraum I, *n* (%)	113 (49,1)	97 (54,2)	0,360
Zeitraum II, *n* (%)	43 (35,8)	78 (55,7)	0,002*

*signifikant

### Todeszeitpunkt

Innerhalb der Subgruppe ereignete sich der Tod sowohl für die Frauen als auch für die Männer am häufigsten (*n* = 125; 35,7 %) in den Wintermonaten (Dezember bis Februar). Am zweithäufigsten (*n* = 81; 23,1 %) trat der Tod in den Sommermonaten (Juni bis August) ein, gefolgt von den Jahreszeiten Frühling (März bis Mai: *n* = 74; 21,1 %) und Herbst (September bis November: *n* = 70; 20,0 %). Im Vergleich beider Untersuchungszeiträume zeigten sich für alle Jahreszeiten keine signifikanten Unterschiede.

In der Vergleichsgruppe trat der Tod ebenfalls am häufigsten (*n* = 93; 29,1 %) in den Wintermonaten ein. Es folgten die Jahreszeiten Frühling in 90 Fällen (28,2 %), Herbst in 69 Fällen (21,6 %) und Sommer in 66 Fällen (20,7 %), wobei sich hier keine signifikanten Unterschiede zwischen beiden Geschlechtern und der Verteilung der Jahreszeiten ergaben. In einem Fall war eine Aussage zum Todeszeitpunkt aufgrund starker Verwesung nicht möglich. Im Vergleich beider Untersuchungszeiträume zeigten sich für alle Jahreszeiten keine signifikanten Unterschiede.

Zwischen der Subgruppe und Vergleichsgruppe ließen sich für die Monate Januar, Februar, April, Mai, Juli, September, Oktober und November keine signifikanten Unterschiede bezüglich des Geschlechts und der erhobenen Zeiträume feststellen. In den Monaten März (*p* = 0,044), Juni (*p* = 0,035) und August (*p* = 0,030) konnten signifikante Unterschiede zwischen der Subgruppe und Vergleichsgruppe erhoben werden. In den Monaten Dezember (*p* = 0,043) und Juni (*p* = 0,046) ließ sich ein signifikanter Unterschied beider Gruppen bezüglich des Zeitraums II feststellen.

### Risikofaktoren kardialer Erkrankungen

Kardiale Erkrankungen sind mit zahlreichen Risikofaktoren wie z. B. dem metabolischen Syndrom assoziiert. Anlässlich einer Obduktion werden Risikofaktoren jedoch nur eingeschränkt erhoben oder sind aus der Vorgeschichte des Verstorbenen nicht hinreichend bekannt. Um Zusammenhänge mit möglichen Risikofaktoren in der Subgruppe zu eruieren, wurden der BMI analysiert sowie die Faktoren Gefäßstatus, Diabetes mellitus und Alkoholabusus ausgewertet. Der Nikotinabusus wurde aufgrund der gering dokumentierten Fallzahl nicht miteingeschlossen.

### BMI

Aufgrund der zentralen Bedeutung von Adipositas für die Entstehung zahlreicher und nicht zuletzt kardiovaskulärer Erkrankungen wurde in der vorliegenden Studie der BMI erfasst.

Innerhalb der Subgruppe zeigte sich eine signifikante Zunahme der Adipositas (Stufe 4) zwischen den Zeiträumen I und II um 11,1 % (Zeitraum I: *n* = 30; 13 %, Zeitraum II: *n* = 29; 24,2 %; *p* = 0,007; phi = −0,153). Mehr Männer als Frauen der Subgruppe wiesen einen BMI oberhalb der Norm und damit mindestens eine Präadipositas auf. Dabei konnten keine signifikanten geschlechtsspezifischen Unterschiede festgestellt werden. Für einen BMI der Stufe 1 traten zwischen beiden Geschlechtern und beiden Zeiträumen keine signifikanten Unterschiede auf.

Innerhalb der Vergleichsgruppe zeigte sich eine nichtsignifikante Zunahme der Adipositas (Stufe 4) zwischen den Zeiträumen I und II um 6,7 %. Zwischen beiden Geschlechtern konnten keine signifikanten Unterschiede für einen BMI oberhalb der Norm (Stufe 3 + 4) erhoben werden. Für einen BMI der Stufe 1 verdeutlichten sich weder signifikante Unterschiede zwischen den Geschlechtern noch zwischen beiden Zeiträumen.

Unterschiede zwischen der Subgruppe und der Vergleichsgruppe sind in Tab. 2 dargestellt.

**Table 2:** 

BMI	Stufe 1 (< 18,5kg/m^2^)	Stufe 2 (18,5 bis < 25,0 kg/m^2^)	Stufe 3 (25,0 bis < 30,0 kg/m^2^)	Stufe 4 (≥ 30kg/m^2^)	Keine Angaben
Subgruppe, *n* (%)	30 (8,6)	131 (37,4)	122 (34,9)	59 (16,9)	8 (2,3)
Vergleichsgruppe, *n* (%)	57 (17,9)	135 (42,3)	67 (21,0)	45 (14,1)	15 (4,7)
*p*-Werte	0,000*	0,135	0,000*	0,460	–
Subgruppe Männer, *n* (%)	13 (6,7)	68 (35,1)	76 (39,2)	33 (17,0)	4 (2,1)
Vergleichsgruppe Männer, *n* (%)	20 (13,9)	67 (46,5)	33 (22,9)	18 (12,5)	6 (4,2)
*p*-Werte	0,037*	0,027*	0,003*	0,361	–
Subgruppe Frauen, *n* (%)	17 (10,9)	63 (40,4)	46 (29,5)	26 (16,7)	4 (2,6)
Vergleichsgruppe Frauen, *n* (%)	37 (21,1)	68 (38,9)	34 (19,4)	27 (15,4)	9 (5,1)
*p*-Werte	0,013*	1,000	0,060	0,960	–
Subgruppe Zeitraum I, *n* (%)	18 (7,8)	96 (41,7)	84 (36,5)	30 (13,0)	2 (0,9)
Vergleichsgruppe Zeitraum I, *n* (%)	35 (19,6)	82 (45,8)	37 (20,7)	20 (11,2)	5 (2,8)
*p*-Werte	0,001*	0,367	0,001*	0,728	–
Subgruppe Zeitraum II, *n* (%)	12 (10,0)	35 (29,2)	38 (31,7)	29 (24,2)	6 (5,0)
Vergleichsgruppe Zeitraum II, *n* (%)	22 (15,7)	53 (37,9)	30 (21,4)	25 (17,9)	10 (7,1)
*p*-Werte	0,210	0,134	0,101	0,312	–

*signifikant

### Gefäßstatus

Da vaskuläre Veränderungen eine große Bedeutung für kardiale Erkrankungen aufweisen, wurden hochgradige Stenosen der zerebralen Gefäße, der Karotiden sowie der thorakalen und abdominellen Aorta erfasst.

Innerhalb der Subgruppe zeigten sich für hochgradige Stenosen der zerebralen Gefäße und Karotiden weder signifikante Unterschiede zwischen den Geschlechtern noch zwischen beiden Zeiträumen. Hochgradige Stenosen der thorakalen Aorta nahmen innerhalb der Subgruppe im Zeitvergleich signifikant um 5,3 % ab (*p* = 0,002; phi = 0,204), zwischen den Geschlechtern gab es keinen signifikanten Unterschied. Auch hochgradige Stenosen der abdominellen Aorta nahmen im Zeitvergleich signifikant ab (*p* = 0,001; phi = 0,206), zwischen den Geschlechtern ließen sich keine signifikanten Unterschiede feststellen.

Ähnliche Ergebnisse konnten in der Vergleichsgruppe erfasst werden. Auch hier ergaben sich keine signifikanten Veränderungen hochgradiger Stenosen der zerebralen Gefäße und Karotiden bezüglich beider Geschlechter und Zeiträume. Eine signifikante Abnahme hochgradiger Stenosen der thorakalen Aorta konnte zwischen den Zeiträumen I und II beobachtet werden (*p* = 0,019; phi = 0,160), nicht jedoch zwischen beiden Geschlechtern. Eine signifikante Abnahme hochgradiger Stenosen zwischen beiden Zeiträumen konnte auch bei der abdominellen Aorta festgestellt werden (*p* = 0,008; phi = 0,173), zwischen den Geschlechtern lag kein signifikanter Unterschied vor.

Die Ergebnisse der Subgruppe und der Vergleichsgruppe sind in Tab. 3 zusammengefasst.

**Table 3:** 

Hochgradige vaskuläre Stenosen bis vollständiger Verschluss	Zerebrale Gefäße	Karotiden	Thorakale Aorta	Abdominelle Aorta
Subgruppe, *n* (%)	35 (10,0)	57 (16,3)	50 (14,3)	104 (29,7)
Vergleichsgruppe, *n* (%)	27 (8,5)	42 (13,2)	34 (10,7)	84 (26,3)
*p*-Werte	0,529	0,306	0,058	0,181
Subgruppe Männer, *n* (%)	21 (10,8)	33 (17,0)	29 (14,9)	58 (29,9)
Vergleichsgruppe Männer, *n* (%)	11 (7,6)	17 (11,8)	12 (8,3)	36 (25,0)
*p*-Werte	0,333	0,226	0,047*	0,213
Subgruppe Frauen, *n* (%)	14 (9,0)	24 (15,4)	21 (13,5)	46 (29,5)
Vergleichsgruppe Frauen, *n* (%)	16 (9,1)	25 (14,3)	22 (12,6)	48 (27,4)
*p*-Werte	1,000	0,926	0,537	0,537
Subgruppe Zeitraum I, *n* (%)	24 (10,4)	41 (17,8)	37 (16,1)	74 (32,2)
Vergleichsgruppe Zeitraum I, *n* (%)	19 (10,6)	19 (10,6)	24 (13,4)	53 (29,6)
*p*-Werte	1,000	0,064	0,121	0,333
Subgruppe Zeitraum II, *n* (%)	11 (9,2)	16 (13,3)	13 (10,8)	30 (25,0)
Vergleichsgruppe Zeitraum II, *n* (%)	8 (5,7)	23 (16,4)	10 (7,1)	31 (22,1)
*p*-Werte	0,307	0,620	0,500	0,790

*signifikant

### Diabetes mellitus

Innerhalb der Subgruppe war in 66 Fällen (18,9 %) die Diagnose eines Diabetes mellitus vermerkt. Bezüglich beider Geschlechter und Zeiträume ergaben sich keine signifikanten Veränderungen.

In der Vergleichsgruppe ließ sich eine ähnliche prozentuale Verteilung feststellen (*n* = 49; 15,4 %). Signifikante Veränderungen bezüglich beider Geschlechter und Zeiträume konnten nicht eruiert werden.

Stellt man die Subgruppe der Vergleichsgruppe gegenüber, so wiesen im Zeitraum I signifikant mehr Personen der Subgruppe einen Diabetes mellitus auf (Subgruppe Zeitraum I: *n* = 45; 19,6 %, Vergleichsgruppe Zeitraum I: *n* = 21; 11,7 %; *p* = 0,033). Es gab keine signifikanten Veränderungen bezüglich der Geschlechter oder des Zeitraums II.

### Alkoholabusus

Die Angaben bezüglich eines Alkoholabusus aus Vorbefunden machten nur eine geringe Fallzahl aus. Auf Basis der vorliegenden Daten konnten folgende Ergebnisse erhoben werden:

Innerhalb der Subgruppe lag in 26 Fällen (7,4 %) ein übermäßiger Alkoholkonsum vor, wobei nur 6 Frauen (3,8 %), jedoch 20 Männer (10,3 %) betroffen waren. Hierbei konnte ein signifikanter Unterschied (*p* = 0,037) zwischen beiden Geschlechtern festgestellt werden, nicht jedoch zwischen beiden Zeiträumen.

In der Vergleichsgruppe wurde in 22 Fällen (6,9 %) ein Alkoholabusus dokumentiert. Auch hier zeigte sich, dass signifikant mehr Männer (Männer: *n* = 15; 7,7 %, Frauen: *n* = 7; 4 %; *p* = 0,042) als Frauen an Alkoholabusus litten. Zwischen beiden Zeiträumen konnten keine signifikanten Veränderungen beobachtet werden.

Zwischen der Subgruppe und der Vergleichsgruppe ergaben sich bezüglich der Geschlechter und Zeiträume keine signifikanten Veränderungen.

### Herzparameter innerhalb der Subgruppe

Ein Myokardinfarkt wurde in 134 Fällen (38,3 %) festgestellt. Hierbei handelte es sich in 48 Fällen (35,8 % aller Myokardinfarkte) um die Erstmanifestation eines akuten Myokardinfarkts und in 34 Fällen (25,4 % aller Myokardinfarkte) um einen Reinfarkt. In 52 Fällen (38,8 % aller Myokardinfarkte) konnten anhand von Narben stattgehabte Myokardinfarkte festgestellt werden, welche im Rahmen der Obduktion nicht als todesursächlich gewertet wurden. Im Vergleich beider Zeiträume zeigten sich keine signifikanten Veränderungen. Erwähnenswert ist, dass der Anteil der Männer (*n* = 85; 43,8 %), welche einen Myokardinfarkt erlitten, signifikant (*p* = 0,024) über dem der betroffenen Frauen lag (*n* = 49; 31,4 %). Die Verteilung der Myokardinfarkte nach Geschlecht und Altersklasse sowie die Lokalisationen der Myokardinfarkte sind in Tab. 4 dargestellt.

**Table 4:** 

	Gesamt	Frauen	Männer
**Myokardinfarkte**			
Altersklasse 1, *n* (%)	57 (75,7)	10 (20,4)	47 (55,3)
Altersklasse 2, *n* (%)	47 (72,6)	20 (40,8)	27 (31,8)
Altersklasse 3, *n* (%)	29 (49,6)	18 (36,7)	11 (12,9)
Altersklasse 4, *n* (%)	1 (2,0)	1 (2,0)	0 (0,0)
**Myokardinfarktlokalisation**			
Vorderwand	42	11	31
Hinterwand	87	34	53
Septum	34	9	25
Seitenwand	16	7	9
Unspezifisch	19	11	8

Es zeigte sich eine signifikante Abnahme der hochgradigen bis verschließenden Koronarsklerosen zwischen den Zeiträumen I und II (Tab. 5)*.* Zwischen beiden Geschlechtern ließ sich kein signifikanter Unterschied feststellen.

**Table 5:** 

Hochgradige vaskuläre Stenosen bis vollständiger Verschluss	Zeitraum I	Zeitraum II	*p*-Werte
RIVA, *n* (%)	165 (71,7)	78 (65,0)	0,029*(phi = 0,128)
RCX, *n* (%)	150 (65,2)	60 (50,0)	0,000*(phi = 0,224)
RCA, *n* (%)	149 (64,8)	62 (51,7)	0,001*(phi = 0,202)

*RIVA* Ramus interventricularis anterior, *RCX* Ramus circumflexus, *RCA* Arteria coronaria dextra

*signifikant

Weitere herzspezifische Befunde sind in Tab. 6 zusammengefasst.

**Table 6:** 

Herzspezifische Befunde innerhalb Subgruppe	Männer	Frauen	*p*-Werte	Zeitraum I	Zeitraum II	*p*-Werte
Herzschrittmacher, *n* (%)	19 (9,8)	12 (7,7)	0,618	19 (8,3)	12 (10,0)	0,730 (phi = −0,029)
Bypass, *n* (%)	28 (14,4)	8 (5,1)	0,008*	22 (9,6)	14 (11,7)	0,668 (phi = −0,033)
Stent, *n* (%)	17 (8,8)	3 (1,9)	0,012*	5 (2,2)	15 (12,5)	0,000* (phi = −0,211)
Herzgewicht ≥ 500 g, *n* (%)	120 (61,9)	50 (32,1)	0,000*	111 (48,3)	59 (49,2)	0,847 (phi = −0,017)
Herznarbe, *n* (%)	191 (98,4)	100 (64,1)	0,000*	193 (83,9)	98 (81,7)	0,702 (phi = 0,029)

*signifikant

## Diskussion

Die Tendenz der zunehmenden Alterung der Gesellschaft zeichnete sich im Zeitvergleich auch innerhalb der Gruppe der natürlich Verstorbenen ab. In Übereinstimmung mit der Literatur [25, 42, 98] machte die Gruppe der natürlich Verstorbenen unter allen Obduzierten den größten Anteil aus. In den vergangenen Jahren konnte ein deutlicher Rückgang kardialer Mortalitätsraten verzeichnet werden [27]. Jedoch nahmen innerhalb der natürlich Verstorbenen kardiale Todesursachen, wie auch im internationalen Vergleich [3, 4, 14, 31, 48, 55, 62, 64, 68, 72, 77, 91, 98], nach wie vor die führende Rolle ein.

Analog zur Literatur [8, 35, 64, 77, 101] überwog das männliche Geschlecht innerhalb der Subgruppe und zeigte auch im Zeitvergleich eine zunehmende Tendenz. Schon Isles et al. [40] beschrieben eine höhere Inzidenz von Herzerkrankungen bei Männern im Vergleich zu Frauen. In ihrer Studie beschrieben Wang et al. [94] eine kardioprotektive Wirkung des weiblichen Geschlechts, so führte oxidativer Stress bei Frauen seltener zum Zelltod als bei Männern. Männer der Subgruppe starben ca. 10 Jahre vor den Frauen, ähnliche Ergebnisse zeigen Isles et al. [40] und Größwald et al. [32].

Im Durchschnitt verstarb die Subgruppe 1,5 Jahre früher als die Vergleichsgruppe. Eine mögliche Erklärung hierfür wäre, dass ein Großteil der Männer (57,2 %) innerhalb der Subgruppe bereits in der ersten Altersklasse verstarb.

Das signifikant häufigere Vorkommen von Bypässen und Stents kardial verstorbener Männer im Vergleich zu den Frauen deckt sich mit den Ergebnissen von Ghali et al. [28]. Außerdem wiesen in dieser Subgruppe, wie bei Turkbey et al. [88], mehr Männer Herznarben auf. Das signifikant häufigere Vorkommen eines kritischen Herzgewichtes von mindestens 500 g [71] bei Männern stimmt mit den Ergebnissen von Molina et al. [58, 59] und weiteren Studien [24, 36, 64, 81] überein.

Törő et al. zeigten in ihrer Autopsiestudie, dass kardiovaskuläre Todesfälle im Alter einer bestimmten saisonalen Verteilung folgen. Das Maximum der Todesfälle trat im Winter (Dezember, Januar) auf und korrelierte negativ mit der saisonalen Temperaturverteilung [85]. Diese Erkenntnisse decken sich mit den Ergebnissen dieser Studie. Auch weitere Studien [11, 17, 57, 86] wiesen eine saisonale Häufung kardiovaskulärer Todesfälle in den Wintermonaten auf. Als mögliche Erklärung bietet Vuori [93] die bei Kälte beeinträchtigte Sauerstoffversorgung des Herzmuskels durch koronare Vasokonstriktion, insbesondere in atherosklerotisch veränderten Gefäßen. Laut Barnett et al. [5] führt Kälteeinwirkung zu kardiovaskulärem Stress, welcher sich in Blutdruckänderungen, Vasokonstriktion und erhöhter Blutviskosität – insbesondere bei älteren Menschen – widerspiegelt. Braga et al. [9] beobachteten eine erhöhte Mortalität bei heißen und kalten Temperaturen. Dabei bestand bei heißen Temperaturen eine erhöhte Mortalität für den akuten Myokardinfarkt. Übereinstimmend zeigten sich in dieser Studie für den Zeitraum II signifikant mehr kardiale Todesfälle für den Sommermonat Juni als in der Vergleichsgruppe.

Adipositas ist einer der Hauptrisikofaktoren für die Entwicklung kardialer Erkrankungen [12, 37, 44, 70, 96]. So zeigte sich auch in dieser Studie, dass signifikant mehr Betroffene – insbesondere Männer – innerhalb der Subgruppe einen BMI oberhalb der Norm (Stufe 3) aufwiesen als in der Vergleichsgruppe. Dies unterstreichen auch Finger et al. [23]. Ebenso ergab sich in dieser Studie eine signifikante Zunahme der Adipositas (Stufe 4) zwischen den Zeiträumen I und II, die sich mit Angaben der WHO [97], Finger et al. [23] und Mensink et al. [56] deckt. Begründet sein könnte dies v. a. durch Bewegungsmangel [12, 19, 61, 70, 96], zunehmend sitzende Tätigkeit und ungesünderen Lebensstil in Wohlstandsregionen.

Die Lebenserwartung Adipöser ist folglich geringer als die von Normalgewichtigen [6, 95]. Neuere Erkenntnisse, beispielsweise von Petrilli et al. [67] und Simonnet et al. [80] zeigen, dass Adipositas auch einen Risikofaktor für die Entwicklung eines schweren Verlaufes einer SARS-CoV-2-Infektion und einer damit verbundenen höheren Mortalität darstellt. Die Subgruppe erfüllte damit wesentliche der zum jetzigen Zeitpunkt bekannten Risikofaktoren und wäre möglicherweise bei einer zum damaligen Zeitpunkt vorliegenden SARS-CoV-2-Pandemie zu einem früheren Zeitpunkt gestorben.

Neben der Koronarsklerose sind auch hochgradige Stenosen der Karotiden und Aorta für schwerwiegende kardiovaskuläre Ereignisse bedeutend, worauf beispielsweise die Studien von Espinola-Klein und Rupprecht et al. [20] sowie Jachau et al. [41] Bezug nehmen. Auch in dieser Arbeit zeigten sich, wenn auch nicht signifikant, mehr hochgradige Stenosen der Karotiden und thorakalen Aorta in der Subgruppe als in der Vergleichsgruppe.

Zhou et al. [100] untersuchten 727 Autopsieberichte von Diabetikern. Die häufigste Todesursache waren mit 55 % kardiovaskuläre Erkrankungen. Auch in dieser Studie lag Diabetes mellitus während des Zeitraums I signifikant häufiger in der Subgruppe als in der Vergleichsgruppe vor. Diese Ergebnisse werden durch weitere Studien [13, 63] belegt.

In der Obduktionsstudie von Laberke et al. [47] bezüglich des Alkoholabusus dominiert deutlich das männliche Geschlecht mit einem Verhältnis von 4,7:1. Ähnliche Ergebnisse zeigten auch Rommel et al. [74]. Übereinstimmend wiesen auch in vorliegendem Studienkollektiv signifikant mehr Männer einen Alkoholabusus auf. Die Bedeutung kardialer Risikofaktoren wurde bereits in der Framingham-Studie [55] dargelegt. Die Ergebnisse der hier vorliegenden Arbeit zu den Risikofaktoren Adipositas, Diabetes mellitus sowie Alkoholabusus decken sich mit Angaben großer Studien, wie der Copenhagen City Heart Study [78]. Jedoch muss einschränkend erwähnt werden, dass anamnestische Angaben in einer Vielzahl der Obduktionen nicht enthalten sind und eine Interpretation nur mit einer gewissen Zurückhaltung möglich ist.

Entgegen den Beobachtungen von Mc Gee et al. [51], in welchen 20,5 % der Frauen und nur 18,4 % der Männer einen akuten Myokardinfarkt erlitten, wiesen in vorliegender Studie signifikant mehr Männer als Frauen einen Myokardinfarkt auf. Dieses Ergebnis deckt sich mit zahlreichen internationalen Studien [2, 14, 16, 32, 39, 43, 75, 91, 101]. Neben William et al. [44] und Mörl et al. [60] zeigte sich noch in weiteren Studien [2, 32, 40, 75], dass Männer in jüngerem Alter einen Myokardinfarkt erlitten als Frauen. Identische Ergebnisse ließen sich auch in dieser Studie erkennen. Bei Frauen manifestierte sich der Myokardinfarkt rund 10 Jahre später. Entscheidende Einflussgrößen könnten hierbei sowohl eine kardioprotektive Wirkung bei prämenopausalen Frauen als auch häufigere Risikofaktoren bei Männern in jüngerem Alter darstellen.

Mc Gee et al. beobachteten in ihrer geriatrischen Autopsiestudie in nur 13 Fällen eine milde Koronarsklerose, während in über 90 % eine mittelschwere bis schwere stenotische Koronarsklerose vorlag [51]. In dieser Studie zeigte sich eine signifikante Abnahme der hochgradigen bis verschließenden Arteriosklerosen in allen 3 Koronarien zwischen den Zeiträumen I und II. Dieses Ergebnis stimmt mit internationalen multizentrischen Studien [46, 78, 87] überein. Eine mögliche Erklärung der Abnahme dieser Koronarverschlüsse bieten die bessere medizinische Versorgung und Intervention z. B. mittels Stents, die Zunahme von Präventionen und regelmäßigen „Check-ups“, ebenso die verbesserten medikamentösen Therapieansätze (z. B. früher Einsatz von Statinen als Dauermedikation). Trotz dieser erfreulichen Entwicklungen stehen kardiovaskuläre Todesursachen noch immer an erster Position [84, 92]. Die Abnahme der Koronarverschlüsse zwischen den Zeiträumen I und II lässt einen Zusammenhang zur signifikant höheren Stent-Implantationsrate zwischen beiden Zeiträumen erkennen. Dieses Ergebnis spiegelt sich auch in größeren Datenerhebungen wider. So führt Deutschland im europäischen Vergleich die meisten Koronarinterventionen durch [33]. Dabei ist die Anzahl der Stent-Implantationen steigend [89, 90].

## Limitationen

In vorliegender Studie handelt es sich um ein rechtsmedizinisches Untersuchungskollektiv. Rechtsmedizinische Obduktionen erfolgen überwiegend auf Veranlassung der Staatsanwaltschaften/Ermittlungsbehörden und dienen der Fragestellung, ob der Verstorbene eines nichtnatürlichen Todes verstorben ist, und ob ein mögliches Fremdverschulden vorliegt [54]. Wird im Leichenschauschein der Verdacht auf eine ungeklärte oder nichtnatürliche Todesart angegeben, kann bzw. muss auf Veranlassung der Ermittlungsbehörden in Abhängigkeit vom Sachverhalt eine Obduktion erfolgen. Wird hingegen eine natürliche Todesart angegeben, so werden die Verstorbenen in der Regel nicht zur Obduktion in das Institut der Rechtsmedizin Frankfurt am Main überführt, da bei einer natürlichen Todesart die Ermittlungsbehörden nicht eingeschaltet werden. Somit besteht bereits aufgrund der Angaben auf dem Leichenschauschein eine Vorselektion durch etwaige Anordnungen der Ermittlungsbehörden. Das vorliegende Untersuchungskollektiv spiegelt somit nur einen geringen Prozentsatz aller an einer natürlichen Todesart Verstorbenen im Einzugsgebiet Frankfurt am Main wider. Die Übertragbarkeit auf die Gesamtbevölkerung im Großraum Frankfurt am Main ist somit eingeschränkt.

Da der Selektionsbias durch die Ermittlungsverfahren die beiden Untersuchungszeiträume gleichförmig betrifft, lassen sich Unterschiede bei den Behandlungsmethoden, wie z. B. die Implantation von Stents im Zeitvergleich gut aufzeigen, sodass vorsichtige Rückschlüsse auf Veränderungen der medizinischen Versorgung gezogen werden können.

Angaben zu den Verstorbenen zu Lebzeiten (Medikamenteneinnahme, Vorerkrankungen wie z. B. Diabetes mellitus, Alkoholabusus, Rauchen) sind im Vergleich zu Patientenakten in der vorliegenden Datenerhebung nur sehr lückenhaft bis gar nicht vorhanden. Wenn bei der Obduktion ein nichtnatürlicher Tod ausgeschlossen werden kann, werden weitergehende Risikofaktoren für einen natürlichen Tod in der Regel nicht im Detail geklärt, sodass die Aussagekraft z. B. hinsichtlich Alkoholabusus oder Diabetes mellitus eingeschränkt ist.

Eine Vergleichbarkeit zwischen den Zeiträumen I und II ist aufgrund der abnehmenden Anzahl an Obduktionen der natürlichen Todesfälle nur eingeschränkt möglich. Liegen Unterschiede vor, so können diese u. a. auf die unterschiedlichen Obduktionsfrequenzen zurückzuführen sein. Liegen keine Unterschiede vor, so hatte die abnehmende Obduktionsfrequenz keinen Einfluss. Zur Interpretation wurde deshalb Pearsons Assoziationskoeffizient phi verwendet und entsprechend nach Cohen bewertet.

## Schlussfolgerung

Bei dem dramatischen Rückgang klinisch-pathologischer Obduktionen liefern rechtsmedizinische Obduktionsstudien inzwischen wertvolle Beiträge nicht nur primär für die Ermittlungsverfahren, sondern auch bei der epidemiologischen Darstellung von Erkrankungen und deren Ursachen. Dies wird aktuell durch die rechtsmedizinischen Obduktionsstudien bei an SARS-CoV-2 Verstorbenen besonders deutlich [15, 18, 45].

Rechtsmedizinische Obduktionsstudien etablieren sich trotz des Selektionsbias zunehmend bei klinischen Fragestellungen als Goldstandard und tragen zum weiteren Verständnis pathomorphologischer Veränderungen, z. B. in der Kardiologie, bei. Der Einfluss von Risikofaktoren für die Entstehung kardialer Erkrankungen (Adipositas) gerade im Vergleich verschiedener Zeiträume lässt sich durch eine retrospektive Mortalitätsstudie mit größeren Fallzahlen gut aufzeigen.

Die sinkenden kardialen Todesursachen im Vergleich vom ersten zum zweiten Zeitraum könnten auf eine bessere medizinische Versorgung zurückzuführen sein. Allerdings stellen kardiale Erkrankungen weiterhin die häufigste Todesursache im rechtsmedizinischen Obduktionsgut bei den Verstorbenen mit einem Lebensalter von 65 Jahren und älter bei der natürlichen Todesart dar.

Die forensische Gerontologie wird im Hinblick auf die natürliche Todesart und deren -ursachen – gerade in Pandemiezeiten – an zunehmender Bedeutung gewinnen. Obduktionen sollten zukünftig vermehrt durchgeführt werden.

## Fazit für die Praxis

- Natürliche Todesfälle nehmen im Zeitvergleich – bei sinkenden Obduktionszahlen – ab, stellen jedoch noch immer die häufigste Todesart im Obduktionsgut des Instituts für Rechtsmedizin Frankfurt am Main der 65-Jährigen und Älteren dar, v. a. wenn auf dem Leichenschauschein „ungeklärt“ angekreuzt war.
- Trotz verbesserter medizinischer Therapieansätze sind kardiale Todesursachen im Obduktionsgut des Instituts für Rechtsmedizin Frankfurt am Main bei älteren Verstorbenen bei der natürlichen Todesart führend.
- Obduktionen bei Verstorbenen mit einem Alter von 65+ sind unerlässlich. Sie tragen bei den bekannten Fehlern bei der Leichenschau zur Klärung der tatsächlichen Todesart und -ursachen bei und helfen, jenseits des Erkenntnisgewinns für die Ermittlungsverfahren, mittels Erhebung pathomorphologischer Befunde unter Berücksichtigung klinischer Symptome Organpathologien und Krankheitsverläufe besser zu verstehen.
- Bei dem allgemeinen Rückgang klinisch-pathologischer Obduktionen leisten rechtsmedizinische Obduktionsstudien (inzwischen durchaus Goldstandard) trotz der genannten Limitationen einen wertvollen Beitrag zum Vergleich medizinischer Versorgung, z. B. in der Kardiologie (Stent) über unterschiedliche Zeiträume.
- Eine generelle Erhöhung der Obduktionsfrequenz könnte zur Steigerung der Qualität in der medizinischen Versorgung beitragen.
- Aufgrund der Alterung der Gesellschaft wird die forensische Gerontologie in der Bedeutung für die Rechtsmedizin eine zunehmende Rolle spielen.
